# Supplementary material for: Chromothripsis during telomere crisis is independent of NHEJ, and consistent with a replicative origin
Source: Genome Res. 2019 May;29(5):737–49. doi: 10.1101/gr.240705.118 (PMC6499312; doi:10.1101/gr.240705.118)
Supplement: Supplemental Material [file supp_gr.240705.118_Supplemental_file_1.zip › contigs/annotated_contigs/DB57/contig.4.DB57_length_771_mean_cov_7.45006485084.docx]

**DB57_length_771_mean_cov_7.45006485084**

```
AAGGGTATTTCATCCACACTGTTTTTTAAATGCTTCCTAGTCAAACTTGAATATGCAATGAGACGCGGGAGCTTTGTGGCGGAAATATA
 >chr7:155264407-155264748 + E=3e-193
ATTAAACTGGGGAAAGATGTAAAAGCTGAAGAATGGGATGACATCAGGCTGATTTCACACTCGGCAGTCGGATCGCTGGGCCCAAGCCG

CGCTCTTGCCACGCAAGGCAGATCAAAGTGCCCTGCCACCGCTAAAAAGGCAAAAGGGGACTTAAGTATGCTAATCCCCAGGACAAATA

TATTTTAATCTTGTTAGAATACAAGTTAATGCTGCAGCTCAGTGGCTGAACTTGGTCAGACTGTAGAGATCCA|T|GGCAGTGGCTATC
                                                                          >chr7:1539354
TTGGTTTGCTGGTCTGAATCATGGGAACAATAGTGCTGCTGTGCTTCCTTGTTAGGCTATCGTGTTACTTAAATCAGACAGTCTAATCC
63-153935673 - E=2e-114 p=3e-02
GACTCTGACTTAACCTCTTAATCCACGCTAGCCAGTTATTGCTATTATCACCTCATATTGTCCTCGATTTCATTTTTAAGGAGAAAAAT

TTTACACCCCTTCTGAA|T|GAGATTGGAAGGTGCTGGGTTTTATTCTGTGAGATTGGAGGTATTGAGTGAGATTGGAAGGTGTTGGAG
                  >chr7:154087508-154087696 + E=3e-101 p=1e-03
AGTTTCAAGCAGAAAAACTGTAAGATCTGCAGATGTTTTAAATAGATGTCTCTGTAAATTGTAAAACTGTAAGACATGGGTAAGAAGCA

ATCATTTTCAGGGGTGAGACTGCTTGTA|G|CACTTTGGATAATGTCTCTGTTGAGTTGCTTTTA                             >chr6:132126871-132126906 + E=3e-0
```
